# Low-Frequency *IL23R* Coding Variant Associated with Crohn’s Disease Susceptibility in Japanese Subjects Identified by Personal Genomics Analysis

**DOI:** 10.1371/journal.pone.0137801

**Published:** 2015-09-16

**Authors:** Kei Onodera, Yoshiaki Arimura, Hiroyuki Isshiki, Kentaro Kawakami, Kanna Nagaishi, Kentaro Yamashita, Eiichiro Yamamoto, Takeshi Niinuma, Yasuyoshi Naishiro, Hiromu Suzuki, Kohzoh Imai, Yasuhisa Shinomura

**Affiliations:** 1 Department of Gastroenterology, Rheumatology, and Clinical Immunology, Sapporo Medical University, Sapporo, Japan; 2 Department of Anatomy, Sapporo Medical University, Sapporo, Japan; 3 Department of Molecular Biology, Sapporo Medical University, Sapporo, Japan; 4 Department of Educational Development, Sapporo Medical University, Sapporo, Japan; 5 Center for Antibody and Vaccine Therapy, Institute of Medical Science, The University of Tokyo, Tokyo, Japan; Universite de Montreal, CANADA

## Abstract

**Background:**

The common disease-common variant hypothesis is insufficient to explain the complexities of Crohn’s disease (CD) genetics; therefore, rare variants are expected to be important in the disease. We explored rare variants associated with susceptibility to CD in Japanese individuals by personal genomic analysis.

**Methods:**

Two-step analyses were performed. The first step was a trio analysis with whole-exome sequence (WES) analysis and the second was a follow-up case-control association study. The WES analysis pipeline comprised Burrows-Wheeler Aligner, Picard, Genome Analysis Toolkit, and SAMTOOLS. Single nucleotide variants (SNVs)/indels were annotated and filtered by using programs implemented in ANNOVAR in combination with identity-by-descent (IBD), subsequently were subjected to the linkage based, and *de novo* based strategies. Finally, we conducted an association study that included 176 unrelated subjects with CD and 358 healthy control subjects.

**Results:**

In family members, 234,067–297,523 SNVs/indels were detected and they were educed to 106–146 by annotation based filtering. Fifty-four CD variants common to both individuals of the affected sib pair were identified. The linkage based strategy detected five candidate variants whereas the *de novo* based strategy identified no variants. Consequently, five candidates were analyzed in the case-control association study. CD showed a significant association with one variant in exon 4 of *IL23R*, G149R [rs76418789, *P* = 3.9E-5, odds ratio (OR) 0.21, 95% confidence interval (CI) 0.09–0.47 for the dominant model (AA + AG versus GG), and *P* = 7.3E-5, OR 0.21, 95% CI 0.10–0.48 for AG versus GG, and *P* = 7.2E-5, OR 0.23, 95% CI 0.10–0.50 for the allele model].

**Conclusions:**

The present study, using personal genomics analysis of a small CD pedigree, is the first to show that the low-frequency non-synonymous variant of *IL23R*, rs76418789, protects against CD development in Japanese subjects.

## Introduction

The IL23 receptor complex consists of IL23R and IL12Rβ1; the latter subunit is common to the IL12 receptor complex, and both of IL23R and IL12Rβ1 are required for IL23A signaling. This IL23 receptor complex associates constitutively with Janus kinase 2 (JAK2), and binds to transcription activator signal transducer and activator of transcription 3 (STAT3) in a ligand-dependent manner. IL23 is essential for maintaining the Th17 response and is associated with Th17 cell lineage differentiation [1]. Moreover, IL23R is expressed on a variety of cells and may directly activate a subset of macrophages, natural killer cells, monocytes, and dendritic cells that secrete IL17 [2].

The *IL23R* gene is located on chromosome 1p31 [3]. The minor allele A of rs11209026 (c.1142G>A, p.Arg381Gln, R381Q) in *IL23R* was shown to be protective against Crohn’s disease (CD) development in the two ethnic cohorts, European [4] and Jewish [5]. In addition to being a major susceptibility gene in inflammatory bowel disease (‘IBD’), *IL23R* is also involved in the pathogenesis of other autoimmune diseases, such as psoriasis [6] and ankylosing spondylitis [7], implicating common proinflammatory pathways in these diseases. Despite these findings, a study in a Japanese population did not find an association between rs11209026 and CD susceptibility; however, this is likely to be because the rs11209026 position was not polymorphic in the Japanese population tested [8]. This discrepancy may also be explained by genetic variants that predispose to CD but that vary between different geographical and racial groups or by rare variants that are common to both the disease and race but that have not been identified by standard genetic methodologies, such as a genome-wide association study [9].

By resequencing of positional candidates, Momozawa et al. reported three new rare *IL23R* variants that protect against CD: p.Arg86Gln (R86Q, rs76575803), p.Gly149Arg (G149R, rs76418789), and p.Val362Ile (V362I, rs41313262). They also presented preliminary evidence that rs76418789 and rs41313262 act protectively against ulcerative colitis [10]. Their results support an increase in effect size with decreasing variant frequency, whereas rare variants explain less of the heritability than their common counterparts do. However, this study confirmed the enrichment of rare variants in at least some genes underlying inherited predisposition to complex diseases. Using next-generation sequencing, Rivas et al. identified significant protective effects against CD of substitutions rs41313262 (*P* = 3.2 × 10^−4^) and rs41313262 (*P* = 1.2 × 10^−5^) in IL23R. This indicated that each of these variants had a protective effect equivalent to that of the more common rs11209026.

In contrast, a Korean study, the first in Asia, identified significant protective effects of rs76418789 (*P* = 0.002) in IL23R [11]. This association was recently confirmed for a Korean population with a genome-wide association study [12]. However, it is noteworthy that rs76418789 was not implicated in CD susceptibility (*P* = 0.57) in China [13]. Therefore, rs76418789 warrants exploration in a Japanese population.

Advances in next-generation sequencing technologies have made it possible for human genomic studies to systematically search for rare disease-contributing genetic variants. Unlike population-based studies, rare disease-contributing variants can be enriched in families, for example in trios (parents and an offspring) or other nuclear families. Based on the above concept, in this study, we explored CD susceptibility in Japanese individuals using whole-exome sequencing (WES) to analyze a small CD pedigree.

## Materials and Methods

All results are presented according to the Strengthening the Reporting of Genetic Associations guidelines [14].

### Study subjects

**Fig 1:**
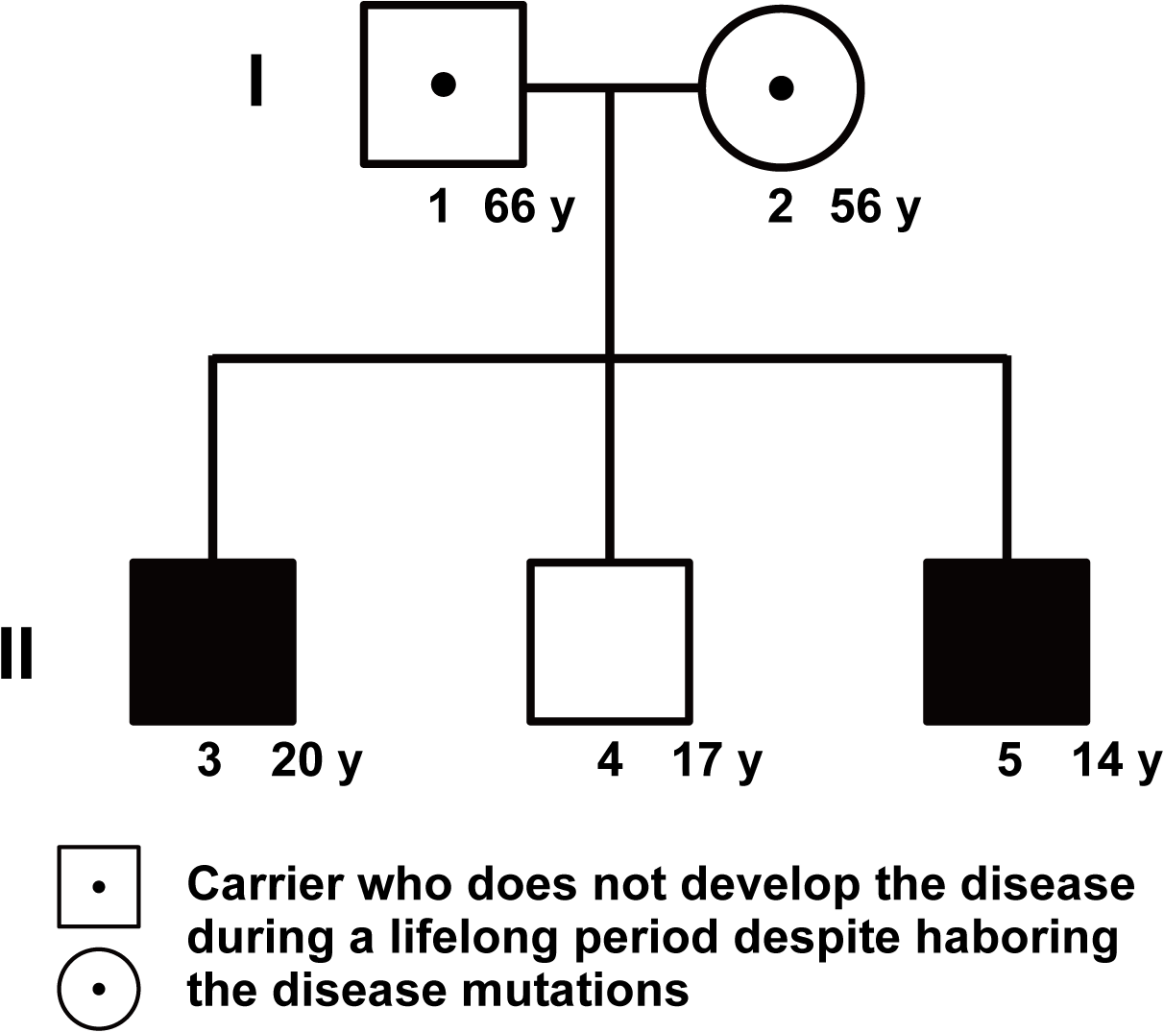
The study pedigree. The pedigree comprised healthy parents (father is indicated as ‘1’ and mother as ‘2’), a healthy second son (4), and older (3) and younger sons (5) affected by Crohn’s disease. Age is indicated in years (y) in the right margin.

**Fig 2:**
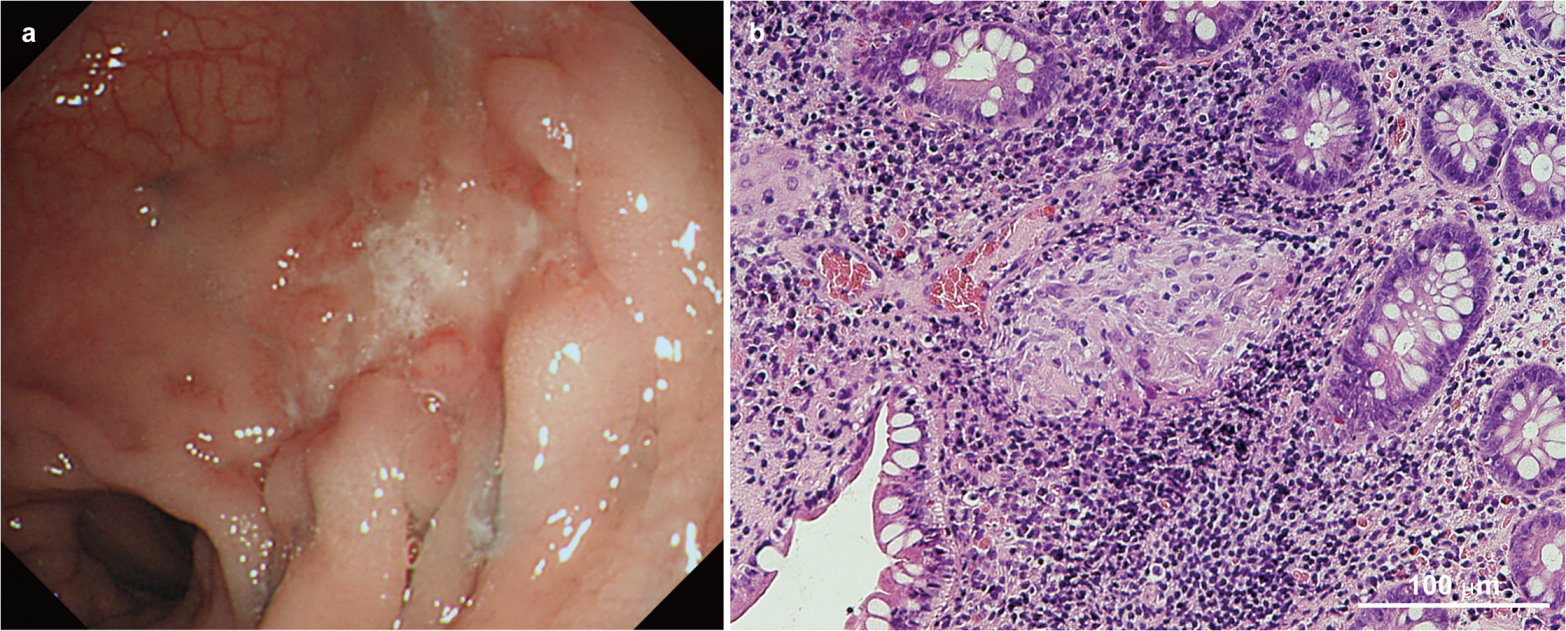
Representative endoscopic and pathological findings of Crohn’s disease in the eldest son. (A) An irregular ulcer encompassed by regenerative epithelia on the transverse colon. (B) Hematoxylin and eosin staining of a typical non-caseating granuloma was seen in the mucosa of biopsy specimens.

A pedigree, comprising a five-member family of healthy parents and three siblings, was recruited to the study. The first and third sons had CD whereas the second son was healthy (Fig 1). Diagnosis of CD was based on standard clinical, radiological, endoscopic, and histological criteria, and intestinal infection was ruled out (Fig 2) [15]. The baseline characteristics of the two affected siblings are shown in Table 1 and are based on the Vienna classification [16].

**Table 1:** Characteristics of the two affected siblings based on the Vienna classification.

Pedigree No.	Age (years)	Gender	Age at diagnosis*	Location**	Behavior†	Ethnicity	Extraintestinal manifestation
**3**	**20**	**M**	**A1 (15 years)**	**L3**	**B1**	**Japanese**	**No**
**5**	**14**	**M**	**A1 (13 years)**	**L3**	**B1**	**Japanese**	**No**

For pedigree number, see Fig 1; For each patient, we determined A (age at diagnosis), L (location), and B (behavior) status according to the Vienna classification as described below.

* Age at diagnosis: A1, <40 years; A2, ≥40 years

** Location: L1, Terminal ileum; L2, Colon; L3 Ileocolon

† Behavior: B1, non-stricturing non-penetrating; B2, Stricturing; B3, Penetrating

Abbreviation: M, male

For follow-up genotyping, an unrelated Japanese population of 179 CD patients (male/female = 116/63, age 16–68 years) and 361 healthy controls (male/female = 156/205, age 19–76) living in Sapporo, Japan were enrolled during a 1.5-year recruitment period. Healthy controls were chosen based on medical history and/or annual check-up records. We investigated the association of five SNPs, prioritized by WES in a parent-offspring trio analysis, with ‘IBD’ susceptibility.

### Ethic Statement

All participants gave their written informed consent, based on the Helsinki Declaration (1964, 1975, amended in 1983, 2003 and 2008) of the World Medical Association. Human Genome, Gene Analysis Research Ethics Committee and the Institutional Review Boards of Sapporo Medical University approved all studies. We obtained informed consent from each family member and furthermore married parents, who exercised parental authority jointly, on behalf of their two minors, the second 17-year-old son and third 14-year-old son.

### Study design for variant prioritization

Two-step analyses were performed. The first step was a trio analysis with WES and the second was a follow-up case-control association study. One of the major challenges in exome analysis is how various variant filtering processes of myriad variant sequence data are precisely interpreted and effectively prioritized to identify susceptibility genes in complex diseases. To this end, we used ANNOVAR (http://www.openbioinformatics.org/annovar/) [17] in combination with statistical genetics approaches, such as identity-by-descent (IBD) and traditional positional mapping, as well as other common approaches reviewed by Gilissen et al. [18] and described below.

**Fig 3:**
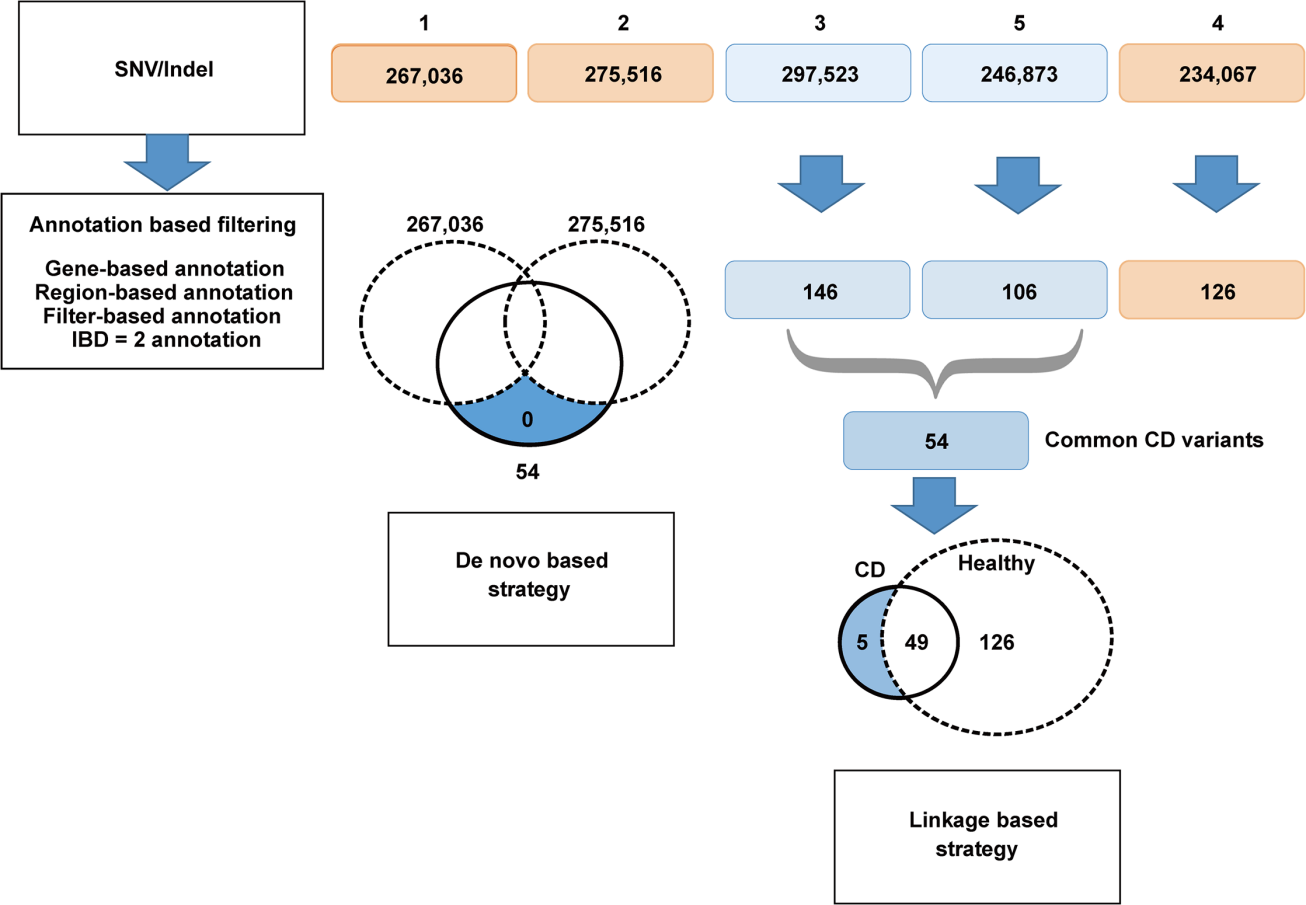
Workflow of whole-exome sequence. Orange rounded rectangles indicate unaffected members, while blue round rectangles indicate affected members. The pedigree numbers, 1, 2, 3, 5, 4, above the round rectangles indicate father, mother, the first, second, and third son, respectively. Numbers inside the rectangles indicate the number of variants. Dashed ellipsoids indicate variants from affected members whereas solid line ellipsoids indicate variants from unaffected members. See text for details.

Candidate pathogenic variants were selected by filtering through gene-based, region-based, and annotation filters using the accessary Perl program, varinats_reduction.pl in ANNOVAR. Gene-based annotation can identify deleterious variants that impair the protein function of genes, while region-based annotation removes repeated sequences, and filter-based annotation excludes common variants registered in either dbSNP, the 1000 Genome project, or the 6500 Exome sequencing project database. However, additional strategies were needed to find the causative mutation among the derived list of approximately 150 variants (Fig 3). For this, assuming the pathogenic variants show an autosomal recessive manner of inheritance in this pedigree, each affected sib inherits the same IBD haplotype from each parent. Because the disease gene must be located within an IBD = 2 region, we used IBD filtering (the R script designated as ibd2.R) developed by Rödelsperger et al. [19]. Furthermore, both traditional positional mapping strategies as well as other common approaches have been adapted for exome sequencing (Fig 3). We briefly explain two of the strategies that we adopted here [18]. Linkage based strategy; we first identified common variants in affected siblings, from which variants in unaffected siblings were subtracted. This means that private variants distinct in the family can be eliminated independent of the mode of inheritance of the disease. *De novo* based strategy; we first identified variants in the affected sibling, from which variants in unaffected parents/siblings were subtracted. This results in identifying *de novo* variants present only in the affected siblings. For detailed information on the whole-exome enrichment and sequencing process and sequence read alignment, variant calling, and annotation, see cited references or URLs. An overview of the workflow of the computational sequence analysis adopted in this study is shown in Fig 3.

### Whole-exome sequence analysis

Genomic DNA from whole blood of study subjects was extracted and with the quality control of the DNA were maintained in a standard manner. The SureSelectXT Human All Exon v5 kit (Agilent Technologies) was used to prepare Illumina sequencing libraries, as reported previously [20] and according to the manufacturer’s instructions. Briefly, 3 μg of high-quality genomic DNA was fragmented with a Covaris S-Series sonicator (Covaris, Woburn, MA, USA). DNA fragments were then end-repaired, and a deoxyadenosine base was added to the 3′ cohesive ends of the fragments. The fragments were then ligated with paired-end adaptors and amplified by polymerase chain reaction (PCR) (five cycles). Amplified adaptor-ligated libraries were then hybridized for 24 h with a biotinylated SuperSelect Oligo Capture library for targeted exomic regions and enriched with streptavidin-conjugated magnetic beads. The enriched libraries were further amplified for 12 cycles with indexed primers and subjected to Illumina sequencing by multiplexing two libraries per lane of the HiSeq 2500 sequencer. The final libraries were denatured with sodium hydroxide and loaded onto an Illumina cBot for cluster generation (cBot User Guide Rev.K), and the primer-hybridized flow cells were then transferred to HiSeq 2500 sequencers for paired-end 100 bp sequencing. The cleaned reads were mapped against UCSC hg19 Genome Reference Consortium Human Reference 37 (GRCh37) (http://genome.ucsc.edu/) using Burrows-Wheeler Aligner (BWA) software (http://bio-bwa.sourceforge.net/). Adapter and linker sequences within reads were identified using the cutadapt program (http://code.google.com/p/cutadapt/) [21] and were removed, along with low-quality reads, by the FASTX-Toolkit (http://hannonlab.cshl.edu/fastx_toolkit/). Thereafter, paired reads were extracted as clean reads for mapping by the cmpfastq_pe program (http://compbio.brc.iop.kcl.ac.uk/software/cmpfastq_pe.php).

### Read alignment, variant calling, and annotation

The Genome Analysis Toolkit (GATK, https://www.broadinstitute.org/gatk/) was used for standardizing binary-sequence alignment format (BAM) files, variant calling with Haplotype Caller, or for correction of a quality scores with Base Quality Score Recalibration. Realignment is recommended because realignment of false-positive single nucleotide variants (SNVs) by, for example, insertions/deletions (indels) can improve a SNV call with high accuracy. Each paired-end read was confirmed to have complementary sequences using the FixMateInformation option in Picard (http://broadinstitute.github.io/picard/). Realigned BAM files were indexed using SAMTOOLS (http://samtools.sourceforge.net/). For tentative SNV calls, quality scores of sequence reads were recalibrated to reflect misread probability using the Base Quality Recalibration option in GATK. By using the PrintReads option in GATK, the above information was added in the order stated to make the BAM file for subsequent SNV/indel calling. The Haplotype Caller in GATK can more accurately detect SNVs/indels compared with the other tools by performing local *de novo* assembly. After detection, variants were added to the annotation using ANNOVAR.

All candidates identified by WES were genotyped by TaqMan chemistry [22], using Custom TaqMan Gene SNP Genotyping Assays except for rs76418789, which was genotyped by both TaqMan chemistry and bidirectional PCR-direct sequencing using a BigDye Terminator Cycle Sequencing Kit with an Applied Biosystems 7500 and 7500 Fast Real-Time PCR systems software (Applied Biosystems, Foster City, CA, USA). Specific primers for SNVs genotyping and sequencing used in the study were shown in S1 Table.

### Genotyping

Follow-up genotyping of 176 subjects with CD and 358 healthy control subjects was performed using TaqMan chemistry [22], using Custom TaqMan Gene SNP Genotyping Assays (Applied Biosystems). Genomic DNA from whole blood of study subjects was extracted and with the quality control of the DNA were maintained in a standard manner.

All samples without template controls were genotyped in duplicate. More than 98% concordance of both members of all duplicate pairs was required. The genotyping of cases and controls was performed simultaneously using the same PCR machine and identical conditions, and scored blind to case-control status. Samples that consistently failed for more than one SNP were removed from the data set prior to calculating call rates. All sample sets genotyped had overall call rates of more than 95%. Genotyping frequencies in unrelated controls were within Hardy–Weinberg equilibrium (HWE).

### Statistical analysis

The HWE was analyzed using Haploview v4.0 [23]. Permutation *P* values for fewer than 10,000 permutations for each search window were calculated as the default [24]. When permutation *P* values were less than 0.05 and more than or equal to the observed *P* values of the test statistic, the permutation test estimates statistical significance avoiding errors from multiple tests. Statistical power of the case-control study was estimated by CaTS version 0.0.2 program (available free from http://www.sph.umich.edu/csg/abecasis/CaTS/) [25]. We used SPSS statistical software 17.0 (Chicago, IL, USA) for other statistical tests. A difference was considered significant when *P* < 0.05 in all two-tailed tests. This study was conducted according to criteria suggested by the NCI-NHGRI Working Group on Replication in Association Studies [26].

## Results

### Workflow of WES in the study pedigree

As depicted in Fig 3, the Haplotype Caller in GATK detected 267,036 SNVs/indels in the father (pedigree number 1), 275,516 in the mother (pedigree number 2), 297,523 in the first son (3), 246,873 in the third son (5), both of whom were affected by CD, and 234,067 in the second unaffected son (4). Each SNV/indel call from the two affected siblings (3 and 5) and unaffected sibling (4) was individually filtered using ANNOVAR and IBD = 2 annotation, which resulted in the retention of 146, 106, and 126 calls, respectively. Next, 54 common CD variants were identified by combining those obtained from pedigree numbers 3 and 5. According to the linkage based strategy, five candidate variants were finally identified by exclusion of the 126 variants from pedigree 4. However, according to the *de novo* based strategy, no *de novo* variants among the common CD variants were identified by excluding all variants derived from the parents (1 and 2). The results obtained from WES were validated by TaqMan Gene SNP Genotyping Assays as shown in S2 Table in detail. Consequently, five candidates were studied in the case-control association study of CD susceptibility.

### Case-control association study of susceptibility to CD

As described in Table 2, we conducted an association study of CD susceptibility in 176 unrelated subjects with CD and 358 healthy control subjects. There was no deviation from the HWE for each variant in the control subjects (data not shown). CD showed a significant association with one variant in exon 4 of *IL23R*, rs76418789 [*P* = 3.9E-5, odds ratio (OR) 0.21, 95% confidence interval (CI) 0.09–0.47 for the dominant model (AA + AG versus GG), and *P* = 7.3E-5, OR 0.21, 95% CI 0.10–0.48 for AG versus GG, and *P* = 7.2E-5, OR 0.23, 95% CI 0.10–0.50 for the allele model]. The allele frequency association was also confirmed with permutation test statistics to adjust for familywise error rate (*P* = 0.0001). Using the CaTS program an adequate statistical power of 83% was confirmed: 0.0000001 as a genome-wide significant level, 0.01 as CD prevalence (the minimum value applied in the program despite a true figure for Japan of 30.3 per 105), 0.08 as disease allele frequency, and 4.35 as genotype relative risk in a dominant inheritance model in 176 CD cases and 358 controls. For the first time, a low-frequency *IL23R* variant, rs76418789, is suggested to act protectively against CD in Japanese individuals.

**Table 2:** Case-control association study of susceptibility to Crohn’s disease.

Gene	rs ID	Genotype	Frequency (%)		*P* value	Permutation *P* value
		Allele	HC	CD		
***IL23R***	**rs76418789**	**G/G**	**299 (83.5)**	**169 (96.0)**	**3.9E-5**	**NA**
		**G/A**	**58 (16.2)**	**7 (4.0)**		
		**A/A**	**1 (0.3)**	**0 (0)**		
		**G**	**656 (91.7)**	**345 (98.0)**	**7.2E-5**	**0.0001**
		**A**	**59 (8.3)**	**7 (2.0)**		
***MLXIP***	**rs3812316**	**C/C**	**268 (74.7)**	**147 (82.6)**	**0.110**	**NA**
		**C/G**	**83 (23.1)**	**29 (16.3)**		
		**G/G**	**8 (2.2)**	**2 (1.1)**		
		**C**	**619 (86.2)**	**323 (90.7)**	**0.038**	**0.178**
		**G**	**99 (13.8)**	**33 (9.3)**		
***AGER***	**rs2070600**	**G/G**	**259 (72.5)**	**114 (65.5)**	**0.218**	**NA**
		**G/A**	**86 (24.1)**	**51 (29.3)**		
		**A/A**	**12 (3.4)**	**9 (5.2)**		
		**G**	**604 (84.6)**	**279 (80.2)**	**0.081**	**0.235**
		**A**	**110 (15.4)**	**69 (19.8)**		
***LOXL1***	**rs3825942**	**G/G**	**249 (72.2)**	**127 (74.7)**	**0.710**	**NA**
		**G/A**	**92 (26.7)**	**42 (24.7)**		
		**A/A**	**4 (1.1)**	**1 (0.6)**		
		**G**	**590 (85.5)**	**296 (87.1)**	**0.506**	**1.000**
		**A**	**100 (14.5)**	**44 (12.9)**		
***HTR4***	**rs10043775**	**T/T**	**183 (68.8)**	**126 (71.6)**	**0.429**	**NA**
		**T/C**	**79 (29.7)**	**45 (25.6)**		
		**CC**	**4 (1.5)**	**5 (2.8)**		
		**T**	**445 (83.6)**	**297 (84.4)**	**0.780**	**1.000**
		**C**	**87 (16.4)**	**55 (15.6)**		

Abbreviations: HC, healthy control; CD, Crohn’s disease; NA, not available

**Fig 4:**
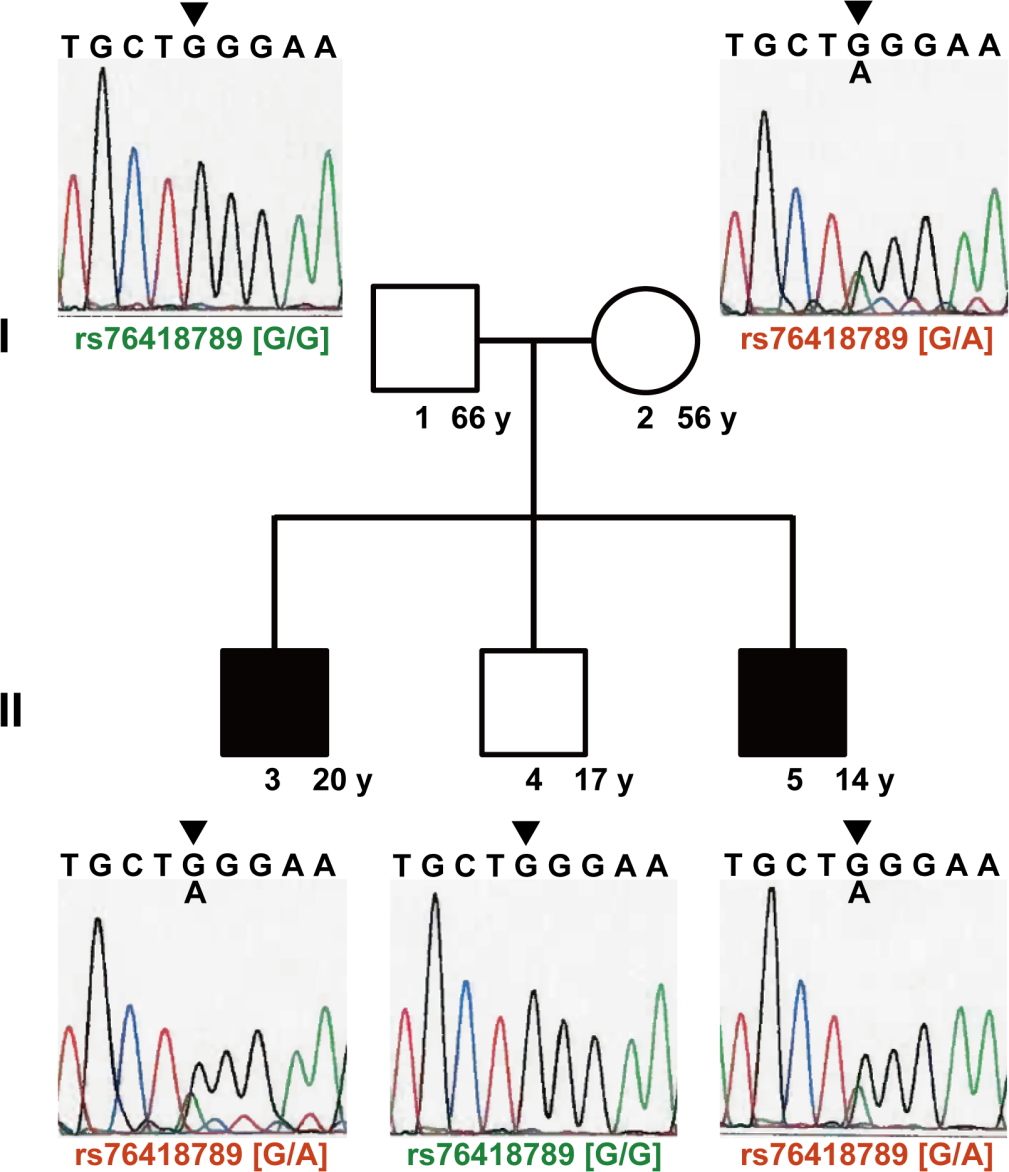
The segregation pattern of rs76418789 in the pedigree. The results from PCR sequencing of rs76418789 were placed on each member in the pedigree. The arrowheads indicated the SNV portion. Red colored variants were derived from the mother’s risk allele, whereas green colored ones were derived from father’s reference allele. The pedigree comprised healthy parents (father is indicated as ‘1’ and mother as ‘2’), a healthy second son (4), and older (3) and younger sons (5) affected by Crohn’s disease. Age is indicated in years (y) in the right margin.

Intriguingly, as depicted in Fig 4, rs76418789 was segregated from the mother to the two affected siblings in this pedigree.

## Discussion

To the best of our knowledge, the present personal genomics analysis of a small Japanese CD pedigree is the first to show that a low-frequency non-synonymous variant of *IL23R*, G149R (rs76418789), protects against CD development in Japanese individuals. Although the results replicate the previous Korean study but not the Chinese study, it is worthwhile to conduct such a replication study in the Japanese population. One variant, R381Q (rs11209026), which is located between the transmembrane domain and the putative JAK2 binding site in the cytoplasmic portion of IL23R, is a loss-of-function variant that reduces STAT3 phosphorylation [27]. rs76418789, in exon 4, is an extremely conserved residue in the extracellular domain of the receptor [10], and is also likely to be loss of function, similar to rs11209026, although this should be confirmed by functional studies. Although we successfully conducted the above strategy to identify a rare CD susceptibility variant, we discuss below whether our success was merely the result of chance.

The benefits of whole genome sequencing can be outweighed by the expense of sequencing multiple subjects and a high false discovery rate; therefore, WES, which targets only expressed genes, is a cost-effective way to explore etiological roles of rare functional variants in complex diseases. Such rare-variant association tests for complex traits are applicable only to population-based or case-control resequencing studies. Relatively few rare-variant association tests for common diseases based on family-based resequencing studies have been published; however, pedigrees possess many attractive characteristics for such analyses. For example, sequencing the parents in trios can identify *de novo* mutations and also allow the study of rare homozygous genotypes, which are difficult to find in population-based designs [28]. Family-based analyses are also attractive because increased genetic load for a disease or trait is often present. Carriers of a minor risk allele are hard to detect in the general population; however, they are more likely to be found in families of probands [29]. Finally, family studies allow the segregation pattern of complex diseases to be studied [30].

Incorporating familial/pedigree information not only provides powerful filtering options for the extensive variant lists that are usually produced by high-throughput sequencing but also allows numerous quality-control steps. For example, this includes the identification of IBD regions, validating the genetic model, and validating haplotype segregation of individuals by making use of IBD information. Two recently published studies describe the above approaches [31,32], which will be vital for families or pedigrees where the genetic model is uncertain because of incomplete penetrance, the presence of possible phenocopy, or where strict Mendelian inheritance does not apply. Taken together, these observations and results suggest that our success was not down to chance but that our approach is valid for identifying rare variants, even for CD in Japanese individuals.

It is not surprising that at some loci with different SNPs, an association is seen in both East Asians and Europeans, albeit in a different disease. For *IL23R*, the primary associated variant in Europeans, rs11209026 was not polymorphic in East Asians, which is consistent with a previous report [8]. However, association with ankylosing spondylitis in East Asians was observed at *IL23R* for a low-frequency non-synonymous SNP, rs76418789 (OR = 1.5, *P* = 8.2 × 10^−4^) [33]. For *IL23R* in CD, rs76418789 was weakly enriched in control cohorts in European decent (*P* = 0.022). A Korean study, the first in Asia, identified significant protective effects of rs76418789 (*P* = 0.002) [11]. This association was recently confirmed for a Korean population with a genome-wide association study [12]. However, it is noteworthy that rs76418789 was not implicated in CD susceptibility (*P* = 0.57) in China [13] whereas rs76418789 was implicated in Japanese CD susceptibility in this study (*P* = 3.9E-5, OR = 0.23). This result may support the notion that most genes conferring susceptibility to IBD were more common between the Japanese and the Korean than the Chinese. Further studies are necessary to confirm the trend in East Asians even in rare variants.

The minor protective allele of rs76418789 was predicted to be deleterious by both SIFT26 and PolyPhen analysis [34], and its frequency (MAF) of rs76418789 was approximately 10 times greater in East Asians than in Europeans (East Asians, 3.7%; Europeans, 0.34%) [35]. From deep sequencing analysis of the Korean genome, rs76418789 showed low linkage disequilibrium (LD) with nearby (<20 kb) SNPs. Because the MAF of rs76418789 in our study was 8.3%, the further cohorts for replication are needed to confirm the association as well as the exact MAF and LD of this region in the Japanese population. Furthermore, although the R script, ibd2.R, used as a filter option seems applicable to WES analysis of two or more siblings affected with an autosomal recessive disease, inheritance of rs76418789 is likely to follow a dominant mode in our study population. To resolve whether using an IBD-based filter is valid in our case warrants further study.

## Conclusions

In conclusion, we have demonstrated through a personal genomics analysis of a small Japanese CD pedigree that a low-frequency protective variant of *IL23R* (G149R, rs76418789) is associated with susceptibility to CD in Japanese individuals. We hope that this more sophisticated approach contributes to resolve the complexity of IBD genetics in Asian populations.

## Supporting Information

S1 Table Specific primers for SNVs genotyping and sequencing used in the study. (DOCX)

S2 Table Validation with TaqMan genotyping assay. (DOCX)
