# Supplementary material for: Low-Frequency IL23R Coding Variant Associated with Crohn’s Disease Susceptibility in Japanese Subjects Identified by Personal Genomics Analysis
Source: PLoS One. 2015 Sep 16;10(9):e0137801. doi: 10.1371/journal.pone.0137801 (PMC4574159; doi:10.1371/journal.pone.0137801)
Supplement: S1 Table — (DOCX) [file pone.0137801.s001.docx]

# S1 Table. Specific primers for SNVs genotyping and sequencing used in the study

| Genes | rs ID | Direction | Sequence |
| --- | --- | --- | --- |
| *IL23R* | rs76418789 | forward | 5′*-ttcacgctgggagctgtaga-*3′ |
|  |  | reverse | 5′*-gaagccgtgaggaagtgacc-*3′ |
| *MLXIP* | rs3812316 | forward | 5′*-ccggactgagtcatggtgaa-*3′ |
|  |  | reverse | 5′*-tgaccacttgaccctggaga-*3′ |
| *AGER* | rs2070600 | forward | 5′*-cagtgtggctcgtgtccttc-*3′ |
|  |  | reverse | 5′*-tgaggccagtggaagtcaga-*3′ |
| *LOXL1* | rs3825942 | forward | 5′*-attcggctttggccaggt-*3′ |
|  |  | reverse | 5′*-cgaaaccctggtcgtaggtc-*3′ |
| *FBXO38* | rs10043775 | forward | 5′*-gccacaaagcatctctgttgat-*3′ |
|  |  | reverse | 5′*-cgccgagtaccgttcttagg-*3′ |
| *GAPDH* | (NM_002046) | forward | 5′*-accacagtccatgccatcac -*3′ |
|  |  | reverse | 5′*-* *tccaccaccctgttgctgta -*3′ |
