# Supplementary material for: Low-Frequency IL23R Coding Variant Associated with Crohn’s Disease Susceptibility in Japanese Subjects Identified by Personal Genomics Analysis
Source: PLoS One. 2015 Sep 16;10(9):e0137801. doi: 10.1371/journal.pone.0137801 (PMC4574159; doi:10.1371/journal.pone.0137801)
Supplement: S2 Table — (DOCX) [file pone.0137801.s002.docx]

# S2 Table. Validation with TaqMan genotyping assay

| Well | Ped ID | rs ID | Allele1 ΔRn | Allele2 ΔRn | Call | Genotype |
| --- | --- | --- | --- | --- | --- | --- |
| A1 | 1 | rs76418789 | 0.44896594 | 2.2232337 | 2/2 Homo | G/G |
| A2 | 2 | rs76418789 | 1.52496552 | 1.31561363 | 1/2 Hetero | G/A |
| A3 | 3 | rs76418789 | 0.11358556 | 0.12268731 | 1/2 Hetero | G/A |
| A4 | 4 | rs76418789 | 0.1812862 | 1.08671832 | 2/2 Homo | G/G |
| A5 | 5 | rs76418789 | 1.75483596 | 1.60548341 | 1/2 Hetero | G/A |
| B1 | 1 | rs3812316 | 1.32548678 | 0.04042667 | 1/1 Homo | C/C |
| B2 | 2 | rs3812316 | 0.91714883 | 1.04772925 | 1/2 Hetero | C/G |
| B3 | 3 | rs3812316 | 1.08863413 | 1.31774926 | 1/2 Hetero | C/G |
| B4 | 4 | rs3812316 | 1.41302443 | 0.02307594 | 1/1 Homo | C/C |
| B5 | 5 | rs3812316 | 0.37068903 | 0.33376491 | 1/2 Hetero | C/G |
| C1 | 1 | rs2070600 | 1.92987561 | 0.23717165 | 1/1 Homo | A/A |
| C2 | 2 | rs2070600 | 1.49373615 | 1.80043852 | 1/2 Hetero | G/A |
| C3 | 3 | rs2070600 | 1.42918134 | 1.7307117 | 1/2 Hetero | G/A |
| C4 | 4 | rs2070600 | 1.11245501 | 0.14737225 | 1/1 Homo | A/A |
| C5 | 5 | rs2070600 | 1.57628369 | 1.86917269 | 1/2 Hetero | A/G |
| D1 | 1 | rs3825942 | 1.57799804 | 0.92392087 | 1/2 Hetero | G/A |
| D2 | 2 | rs3825942 | 1.5232271 | 1.6183362 | 2/2 Homo | G/G |
| D3 | 3 | rs3825942 | 2.01203918 | 1.18688273 | 1/2 Hetero | G/A |
| D4 | 4 | rs3825942 | 1.40333939 | 1.53773928 | 2/2 Homo | G/G |
| D5 | 5 | rs3825942 | 1.66205335 | 1.01683867 | 1/2 Hetero | G/A |
| E1 | 1 | rs10043775 | 2.09305382 | 3.70906782 | 1/2 Hetero | T/C |
| E2 | 2 | rs10043775 | 0.33101523 | 1.77996683 | 2/2 Homo | T/T |
| E3 | 3 | rs10043775 | 1.19419074 | 1.97114038 | 1/2 Hetero | T/C |
| E4 | 4 | rs10043775 | 0.44343299 | 2.8856864 | 2/2 Homo | T/T |
| E5 | 5 | rs10043775 | 1.07167053 | 1.75378871 | 1/2 Hetero | T/C |
